# Body appreciation and body appearance pressure in Norwegian university students comparing exercise science students and other students

**DOI:** 10.1186/s12889-021-10550-0

**Published:** 2021-03-19

**Authors:** Christine Sundgot-Borgen, Jorunn Sundgot-Borgen, Solfrid Bratland-Sanda, Elin Kolle, Monica Klungland Torstveit, Kethe M. E. Svantorp-Tveiten, Therese Fostervold Mathisen

**Affiliations:** 1 Norwegian School of Sport Sciences, Department of Sports Medicine, Sognsveien 220, N-0806 Oslo, Norway; 2 Regional Department for Eating Disorders, Division of Mental Health and Addiction, Oslo University Hospital, Postbox 4956 Nydalen, 0424 Oslo, Norway; 3 Department of Sports, Physical Education and Outdoor Studies, University of Southeast Norway, P.O. Box 235, N-3603 Kongsberg, Norway; 4 University of Agder, Faculty of Health and Sport Sciences, Postbox 422, 4604 Kristiansand, Norway; 5 Østfold University College, Department of Health and Welfare, PO 700, 1757 Halden, Norway

**Keywords:** Body image, Body appreciation, Body appearance pressure, Exercise science, Physical activity, University students, Education, Mental health

## Abstract

**Background:**

Body image is considered a core issue for public health and associates with university students’ overall health. Due to positive associations between exercise and body image, it has been suggested that students within an exercise science program might be more body appreciative compared to other students. On the other hand, the strong societal idealization of the athletic body may expose them to a pressure of having a specific body appearance, which may result in unfavorable health consequences. Nevertheless, studies investigating these hypotheses are lacking. We therefore aimed to explore the level and associations of body appreciation, body appearance pressure, body image related mental health constructs, physical activity, and exercise, in Norwegian university exercise science and non-health reference students.

**Method:**

Male and female exercise science students (*n* = 517) and reference students (*n* = 476), from nine large universities in Norway took part in this cross-sectional study. Participants responded to the Body appreciation scale-2, a self-developed questionnaire on body appearance pressure, Physical Appearance Comparison Scale-Revised, Sociocultural attitudes towards appearance questionnaire-4R, Rosenberg Self-Esteem Scale, Satisfaction with Life Scale, and questions about physical activity and exercise. Between group differences were analyzed using student independent t-test and ANCOVA for parametric data and Chi-square test for categorical data, and associations were evaluated by Person’s correlation. *P*-values ≤0.05 were defined as significant.

**Results:**

Female exercise science students had higher scores on body appreciation compared to reference students. No group difference was found in males. A high percentage of students reported experiencing body appearance pressure, with 69 and 85% among male and female exercise science students, and 57 and 83% among male and female reference students. Fitness centers were the settings where most respondents experienced body appearance pressure. Body appreciation was strongly associated with favorable scores on body image related mental health constructs, while personally experienced body appearance pressure associated with destructive scores on the same constructs.

**Conclusion:**

Body appearance pressure is an issue among university students regardless of study program. Actions to promote body appreciation and prevent body appearance pressure may include an implementation of media literacy, body functionality, and exercise as topics within the education program to safeguard students’ health and well-being.

**Clinical trial registry:**

No. NCT04256967.

**Supplementary Information:**

The online version contains supplementary material available at 10.1186/s12889-021-10550-0.

## Background

Body image has been acknowledged as an important public health issue [1], and different aspects of body image have been reported to associate with the physical, mental, and social health of young adults in higher education [2, 3]. More specifically, women and men who are appreciative towards their body might be more prone to engage in behaviors that are health promotive, making body appreciation important to overall health [4, 5]. Body appreciation is defined as accepting-, holding favorable opinions toward-, and respecting one’s body, resisting the sociocultural pressures to internalize the stereotyped beauty standards and appreciating the functionality and health of the body [5]. As for other body image constructs, there is a gender difference, where males tend to show a stronger body appreciation compared to females [6].

One group that is believed to hold a strong body appreciation is university students within an exercise science study program. This assumption is based on several characteristics related to their study program and education environment that in theory is believed to facilitate body appreciation [7]. Exercise science students are believed to be highly physically active, which positively associates with positive body image [8]. Additionally, their education exposes them to a regular emphasis on body functionality rather than body appearance [9]. Also, their evidence-based knowledge about the relationship between exercise, nutrition, body composition and health, may make them more media literate in relation to body appearance related information on exercise and nutrition trends. This is believed to increase their ability to withstand body image threats [7].

Despite these positive assumptions about exercise science students, previous studies have not investigated whether exercise science students hold a stronger body appreciation compared to other students. One study of male and female fitness instructors [10] reported slightly higher scores on body appreciation compared to scores reported in another study among female undergraduate students [11]. Taking into consideration the difference in study sample, this finding could strengthen the hypothesis that differences might exist between individuals within an exercise- and non-exercise-focused environment.

In contrast to the proposed benefits from being an exercise science student, experiencing body appearance pressure (BAP) has been described as a potential threat to these students’ body appreciation. When body figure idealization is reinforced by an experienced expectation to comply with a certain look, BAP might occur, and can become an important aspect of self- and body evaluation [10]. BAP is a highly discussed phenomenon in the Scandinavian society, but scarcely scientifically measured [12]. In contrast to body appreciation, existing reports show that BAP has been related to reduced quality of life and increased mental health issues among Norwegian youth [13]. Findings among Norwegian high school students indicate that 70% experience BAP, and girls hold the highest prevalence [13]. Therefore, BAP as a new phenomenon, is recognized by the Norwegian government as a risk to young people’s body image and mental health, and a topic included in governmental strategies [12].

In relation to exercise science students, they are exposed to body-oriented curriculum and lectures. Their fellow-students are often characterized with a stronger physique compared to the general student population, and these students take part in numerous of practical sessions within a campus fitness center or sports facilities. Being exposed to these factors combined over a prolonged period of time, might among some students facilitate a preoccupation with healthy eating, exercise, and a need to develop an athletic body type. If students perceive that their skills, academic competence and credibility should be reflected by their body appearance, they might also believe that they will be evaluated based on their body appearance. Using Bourdieu’s concept of capital to explain this phenomenon, exercise science students are then experiencing what can be understood as bodily capital [14, 15]. If this is true, their physical appearance will influence their employment and income from their exercise business [14]. This type and intensity of body focus may result in experiences of BAP and could threaten their level of body appreciation.

Previous studies have reported on such issues. One study reported higher percentage of male and female exercise science students with symptoms of orthorexia nervosa (e.g. an unhealthy relationship with body weight and shape, eating habits, and sometimes exercise) (84.5%) compared to business students (65.4%) [16]. Another study reported a higher frequency of male and female health and physical education teacher students with negative body image and use of unhealthy exercise and eating strategies to control weight, compared to control students [17, 18]. However importantly, these studies did not investigate whether having a strong body appreciation could protect against such challenges.

There is a lack of studies investigating body appreciation and whether BAP is prevalent in the exercise science student population. There is also a lack of studies investigating whether these constructs associate with other health related measures such as body image related mental health constructs, physical activity, and exercise. Hence, a more exploratory study is needed to clarify the role of body appreciation and BAP among exercise science students compared to a reference group, and to further identify whether there is a need for interventions that could safeguard students’ health and well-being.

Therefore, the aims of this study were to explore 1) body appreciation and 2) the experiences of BAP, in Norwegian university students of exercise science programs compared with a reference group. We also aimed to explore 3) the relationship between body appreciation, BAP, body image related mental health constructs, such as physical appearance comparison, internalization, self-esteem, satisfaction with life, and physical activity and exercise, in male and female university students.

## Method

### Study design, procedure, and data collection

This is a cross-sectional study among students attending various universities in Norway, conducted during the period of January–June 2020. Nine universities from large cities in Norway were asked to participate in the study, representing higher education campuses in all cardinal areas in Norway. After consent from the Dean at the relevant faculties of each university, students were informed about the study and asked to participate through their student e-mail and web-based learning management systems. Prior to answering the questionnaire, students were informed that the overall aim of the study was to explore body image and BAP among University students in Norway. Students were then asked to respond to an electronic questionnaire at one time-point, and to answer questions about demographics, body appreciation, BAP, body image related mental health, physical activity, and exercise.

### Participants

Eligibility criteria were male and female students who were fluent in Norwegian. Participants represented either students enrolled in a bachelor or master’s degree in exercise sciences or the reference group who were studying teaching, engineering, or business and administration. Among the 5344 students who were informed about the study, 517 (males *n* = 220, females *n* = 297) exercise science students and 476 (males *n* = 113, females *n* = 363) reference students participated. Among all students, all academic years were represented; 1st year bachelor (35%), 2nd year bachelor (23%), 3rd year bachelor (29%), 1st year masters (8%), and 2nd year masters (5%). The mean (sd) age of the total sample was 24.3 (5.6) years, where female exercise science students were younger than their reference students (*p* = .028). The mean BMI of the total sample was 24.3 (3.7), where we observed a lower BMI in male (*p* = .048) and female (*p* = .004) exercise science students compared to reference students. Among exercise science students, 7 and 6% of male and female students, respectively, reported an immigration background. Among reference students, the prevalence was 13 and 12% in male and female students, respectively. The prevalence was significantly higher among female reference students compared to female exercise science students (*p* = .006).

### Questionnaire

#### Demographics

Students self-reported age, gender, height and weight, academic year, specified their scientific study program, and reported immigration status, where having two parents immigrated defined a student with immigration background.

#### Outcome measures

Body Appreciation Scale-2, Physical Appearance Comparison Scale-Revised, and Sociocultural Attitudes Towards Appearance Questionnaire 4R, have been evaluated on comparable samples but not a Norwegian student sample. Hence, a back-and forth translation from English to Norwegian and Norwegian to English was conducted in collaboration with a person fluent in both English and Norwegian. We further conducted confirmatory (CFA) and exploratory (EFA) factor analyses to evaluate the scales in our sample.

##### Body appreciation scale, version 2 (BAS-2)

BAS-2 measures body appreciation through 10-items, where participants respond to a Likert scale ranging from 1 (*never*) to 5 (*always*), with a higher average score indicating a higher level of body appreciation [5]. The CFA found a good model fit for the BAS-2 in our sample (*x*^*2*^(169) = 35, *p* = <.001, CFI = 0.98, RMSEA = 0.065). In the present study sample Cronbach’s alpha (α) was .95 in both male and females respectively, which is slightly higher than previously reported in young Scandinavian male and females [6].

##### Self-developed questionnaire about body appearance pressure

We measured different aspects of experienced BAP through a self-developed questionnaire. For this study we included questions which asked 1) to what degree students experienced BAP in general (general BAP) 2) to what degree they personally experienced BAP (personal BAP), and 3) experienced BAP in academic and exercise settings. All questions were responded to on a Likert-scale ranging from 1 (*not at all*) to 4 (*to a very high extent*) (Additional file 1). Results are presented as a mean score on each single item in ANCOVA and correlation analyses, or as percentage of students answering “to a high” or “very high” extent, or not, on each single item in Chi-square-tests.

##### Physical appearance comparison scale-revised (PACS-R)

The 11-item PACS-R was used to measure participants’ tendency to compare his or her physical appearance to the physical appearance of others, using a 5-point Likert scale ranging from 0 (*never*) to 4 (*always*), where a higher score indicates a higher engagement in comparison [19]. The CFA found an acceptable model fit for the PACS-R in our sample (*x*^2^(691) = 44, *p* = <.001, CFI = 0.94, RMSEA = 0.12). In the present sample α was .95 and .96 in male and females respectively, which is similar to previous findings in college women [19].

##### Sociocultural attitudes towards appearance questionnaire 4R (SATAQ-4R)

To assess internalization of body ideals, we used the internalization sub-scales of Thin/low body fat internalization, Athletic/muscular internalization, and General attractiveness internalization in the SATAQ-4R [20]. Participants responded to a 5-point Likert-scale ranging from 1 (*strongly disagree*) to 5 (*strongly agree*), where negatively worded items were reversed and a mean score for each sub-test was calculated. A higher score indicates higher degree of internalization. The CFA found a good model fit for the SATAQ-4R sub-tests in our males (*x*^*2*^(25) = 17, *p* = <.001, CFI = 0.99, RMSEA = 0.04), and females (*x*^*2*^(440) = 87, *p* = <.001, CFI = 0.92, RMSEA = 0.08). In the present study, α was .79–.87 and .81–.89 for male and females, respectively. This is similar to reported α in college men and women [20].

##### Rosenberg self-esteem scale

The Rosenberg Self-Esteem Scale (RSES) [21] measures global self-worth where respondents answer 10-items on a Likert-scale ranging from 4 (*strongly agree*) to 1 (*strongly disagree*). Negative worded items were reversed, so that a total score ranges from 10 to 40, where a higher score represents a higher global self-worth. The α in the present study represented an internal consistency of 0.87 and 0.89 for males and females respectively, which is similar to what was reported among Norwegian adults [22].

##### Satisfaction with life scale (SWLS)

The SWLS is a 5-item scale measuring the participants’ perception of their life satisfaction [23]. Responses were rated on a Likert scale ranging from 1 (*strongly disagree*) to 7 (*strongly agree*). The α in the present study sample was .90 in both male and female students, which is similar to previous findings in Norwegian men and women [24].

##### Physical activity and exercise

Students rated, in hours and minutes, their level of physical activity during the last week. Physical activity was defined in the questionnaire as “all bodily movement that lead to an increase in body temperature, and light-heavy shortness of breath” [25]. Students who reported being physically active 150 min or more per week met the current physical activity recommendations [26]. Students also reported number of exercise sessions during the last week, where exercise was defined as “a type of physical activity conducted to maintain or improve physical fitness (e.g. resistance training, cardio). Exercise is more planned than regular physical activity” [25]. Data on physical activity and exercise were treated as continuous data in analyses.

### Statistical analyzes

To evaluate the validity of instruments on our sample, CFA with the maximum likelihood method was conducted in SPPS AMOS 27. All other data were analyses with IBM SPSS version 26. After visually evaluating the data for normality, between group differences were analyzed using student independent t-test for parametric data and Chi-square test for categorical data. ANCOVA was additionally conducted to investigate differences between groups while controlling for age and BMI. These results are only presented in text. The association between variables was evaluated by Person’s correlation and estimated 95% confidence interval from bootstrapping with 1000 replications (for values on r and 95%CI, see Table 3). To explore associations with body appreciation and BAP, we merged exercise science and reference students. Due to suggested differences between males and females in body appreciation and BAP, analyses were split by gender. Data are presented as N (%), mean (sd), mean differences (95%CI), and *p*-values at ≤0.05 were evaluated as statistically significant.

### Ethics

The study was conducted according to the World Medical Association Declaration of Helsinki. It was approved by the Norwegian Regional Committees for medical and health research ethics (No. 33532) and the Norwegian Centre for Research data (No. 978522) and was registered prospectively in the Clinical Trial registry (No. NCT04256967). The students consented to participate by responding to an email containing study information and a letter of informed consent. They accepted by pressing “yes” to the question of consent and were redirected to the online questionnaire which was developed through the web-based system SurveyXact 8.2 offered by Ramböll, Aarhus, Denmark.

## Results

### Body appreciation

First, we analyzed the differences in body appreciation between exercise science and reference students. We found that in females, exercise science students reported higher body appreciation compared to reference students, which remained after controlling for age and BMI (F(1, 598) = 19.81, p = <.001). We found no group difference in males (Table 1).

**Table 1:** Demographics, body image related mental health constructs, physical activity, and exercise in students

	Male								Female					
	Exercise science		Reference					Exercise science		Reference				
	*N*	M (SD)	*N*	M (SD)	Mean diff. [CI 95%]	*p*	d/φ *	*N*	M (SD)	*N*	M (SD)	Mean diff. [CI 95%]	*p*	d/φ *
Age	220	24.56 (4.41)	113	24.66 (5.60)	−0.10 _[−1.20, 1.00]_	.869	–	297	23.58 (6.99)	363	24.36 (5.25)	−0.78 _[−1.50, − 0.06]_	**.028**	0.13
BMI kg/m^2^	220	24.25 (2.59)	113	25.14 (4.34)	−0.89 _[−1.64, − 0.14]_	**.048**	0.25	297	23.48 (2.82)	363	24.40 (5.20)	−0.91 _[−1.57, − 0.26]_	**.004**	0.22
BAS-2	213	3.96 (0.76)	99	3.88 (0.23)	0.07 _[−0.11, 0.26]_	.434	–	280	3.72 (0.77)	322	3.40 (0.83)	0.32 _[0.20, 0.45]_	**<.001**	0.40
Comparison	213	1.40 (0.91)	100	1.22 (0.90)	0.19 _[−0.03, 0.40]_	.090	–	280	1.77 (0.96)	328	2.04 (0.99)	−0.27 _[− 0.43, − 0.11]_	**.001**	0.28
*SATAQ-Athletic*	213	3.14 (0.97)	98	3.04 (0.93)	0.11 _[−0.12, 0.34]_	.367	–	279	3.36 (0.81)	320	2.81 (0.93)	0.55 _[0.41, 0.69]_	**<.001**	0.63
*SATAQ-Thin*	213	2.22 (0.99)	98	2.31 (0.94)	−0.10 _[−0.33, 0.14]_	.427	–	279	4.01 (0.60)	320	4.14 (0.61)	−0.13 _[− 0.22, − 0.03]_	**.012**	0.21
*SATAQ-General*	213	3.45 (0.97)	98	3.30 (0.94)	0.15 _[−0.08, 0.38]_	.212	–	279	2.69 (0.98)	320	3.03 (1.05)	−0.33 _[− 0.50, − 0.17]_	**<.001**	0.33
Self-esteem	213	31.33 (5.07)	96	30.81 (5.61)	−0.52 _[−1.75, 0.79]_	.420	–	279	29.79 (5.25)	316	28.20 (5.85)	1.52 _[2.51, 0.68]_	**.001**	0.27
SWLS	213	23.78 (5.7)	96	23.03 (7.17)	0.75 _[−0.89, 2.40]_	.367	–	279	22.95 (6.09)	314	21.34 (6.59)	1.61 _[0.59, 2.63]_	**.002**	0.25
PA, h/wk.	213	10.40 (5.66)	94	6.40 (5.93)	4.00 _[2.60, 5.40]_	**<.001**	0.07	276	9.48 (6.20)	312	5.16 (4.64)	4.32 _[3.44, 5.20]_	**< .001**	1.31
Exercise sessions/wk.	213	6.70 (2.67)	94	4.41 (2.43)	2.29 _[1.65, 2.92]_	**<.001**	0.90	277	6.50 (2.65)	312	4.09 (1.19)	2.41 _[2.04, 2.78]_	**<.001**	1.67
Meeting PA rec. (%)	213	205 (96.2%)	94	78.7%	74 (17.5%)	**<.001**	0.28*	277	96.4%	312	79.2%	17.2%	**<.001**	0.26*

*Note:* Table shows mean, prevalence and mean differences between male and female student groups. Reference = reference students. Mean diff. = mean difference between exercise science and reference group. Age = years of age. BMI = body mass index (kg/m^2^). BAS-2 = Body appreciation scale-2. SWLS = Satisfaction with life scale. SATAQ-4R = Social attitudes towards appearance questionnaire-4 revised. Comparison = physical appearance comparison. CI = confidence interval. PA h/wk. = physical activity hours per week. Meet PA rec = meet physical activity recommendations of 150 min per week. A *p*-value of ≤0.05 is set as statistically significant when comparing two groups. *d*: Cohen’s *d* and φ*: Phi-coefficient are only presented where there is a significant group difference

### Body image related mental health outcomes

We then analyzed the differences in body image related mental health outcomes between exercise science and reference students. We found that female exercise science students reported more favorable scores on physical appearance comparison (F(1, 604) = 8.51, *p* = .004), more internalization of the athletic body ideal (F(1, 595) = 54.34, *p* = <.001), less internalization of the thin body ideal (F(1,595) = 7.73, *p* = .006) and general attractiveness (F(1,595) = 16.87, *p* = <.001), higher satisfaction with life (F(1,589) = 7.82, *p* = .005), and self-esteem (F(1, 591) = 9.39, *p* = .002), compared to reference students (Table 1). All differences remained after controlling for age and BMI. We found that male exercise science students reported more favorable scores on physical appearance comparison, compared to reference students, which remained after controlling for age and BMI (F(1, 309) = 4.64, *p* = .032) (Table 1).

### Body appearance pressure

#### General and personal experienced body appearance pressure

We analyzed the prevalence of experienced BAP. We found that a general experience of BAP was experienced by most of the students (57–85%). We then analyzed the differences in experienced BAP between exercise science and reference students. We found that general experience of BAP was reported by a higher percentage of male exercise science students (69%) compared to male reference students (57%) (*p* = .037) (Table 2). When treating BAP as continuous data, the difference was still significant when controlling for age and BMI F(1, 324) = 7.93, *p* = .005. Within the total sample, more students experienced a general BAP compared to the number experiencing personal BAP (Table 2).

**Table 2:** The experience of body appearance pressure in academic and exercise settings by male and female university students

	Male				Female			
	Exercise science (*n* = 218)	Reference (*n* = 110)			Exercise science (*n* = 293)	Reference (*n* = 353)		
	n (%)	n (%)	*p*	*φ*	n (%)	n (%)	*p*	*φ*
General experienced BAP	151 (69%)	63 (57%)	**.031**	0.12	248 (85%)	292 (83%)	.511	
Personal experienced BAP	37 (17%)	12 (11%)	.146		84 (29%)	121 (34%)	.127	
Lectures	3 (1%)	3 (3%)	.389		10 (3%)	9 (3%)	.518	
Fellow students	21 (10%)	9 (8%)	.667		38 (13%)	43 (12%)	.763	
Professors	0 (0%)	2 (2%)	**.046**	0.11	4 (1%)	5 (1%)	.956	
Fitness center at campus	40 (19%)	21 (23%)	.530		64 (23%)	110 (38%)	**<.001**	0.17
Commercial fitness center	57 (31%)	21 (26%)	.389		87 (33%)	136 (46%)	**.001**	0.14

Note: Data are presented as number and percentage of students answering “to a high” or “very high” extent to questions on experienced BAP. Exercise science = exercise science students. Reference = reference students. BAP = body appearance pressure. φ* = Phi-coefficient and is only presented where there is a significant group difference. A *p*-value of ≤0.05 is set as statistically significant when comparing two groups. For further explanation of categories, see supplementary file

#### Body appearance pressure in academic and exercise settings

Analyzes were also conducted to investigate students’ experience of BAP in academic and exercise related settings. We found that fellow students, campus fitness centers, and commercial fitness centers stood out as specific settings where personal BAP was experienced (Table 2). We then analyzed the difference between exercise science and reference students. We found that a lower percentage of female exercise science students compared to female reference students, experienced a high level of BAP at fitness centers at campus (23% vs. 38%, *p* = <.01) and commercial fitness centers (33 vs. 46%, *p* = .001). This was also observed when variables were treated as continuous data and controlled for age and BMI for the two settings, respectively (F(1, 642) = 39.60, *p* = <.001, F(1, 642) = 15.51, *p* = <.001).

### Associations between body appreciation, body appearance pressure, body image related mental health constructs, physical activity, and exercise

**Fig. 1:**
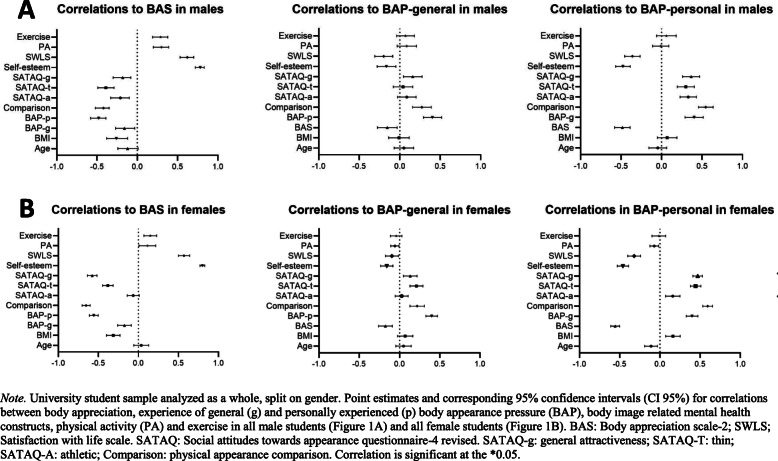
Point estimates (95%CI) for correlations between constructs in male and female university students

First, we performed correlation analyzes to evaluate the associations between body appreciation and other constructs in males and females. We found that body appreciation was moderately to strongly negatively correlated with personal experience of BAP, physical appearance comparison, internalization of the thin body ideal in males and females, and general attractiveness internalization and BMI in females. We also found that body appreciation was moderately to strongly positively correlated with satisfaction with life and self-esteem, in males (Table 3, Fig. 1a) and females (Table 3, Fig. 1b).

**Table 3:** Correlations between variables in male and female university students

	BAS	BAP general	BAP personal
**Male students**			
BAS-2	1	−.16* (−0.27, − 0.03)	−.48* (− 0.58, − 0.39)
Age	−.12* (− 0.24, 0.01)	.05 (− 0.07, 0.17)	−.04 (− 0.16, 0.06)
BMI	−.26* (− 0.38, − 0.12)	.00 (− 0.14, 0.11)	.09 (− 0.06, 0.18)
General experience of BAP	−.16* (− 0.27, − 0.03)	1	.43* (0.28, 0.50)
Personal experience of BAP	−.48* (− 0.58, − 0.39)	.43* (0.28, 0.50)	1
Comparison	−.42* (− 0.52, − 0.35)	.27* (0.16, 0.39)	.54* (0.46, 0.64)
SATAQ-Athletic	−.21* (− 0.33, − 0.10)	.09 (− 0.03, 0.20)	.33* (0.23, 0.43)
SATAQ-Thin	−.39* (− 0.49, − 0.29)	.04 (− 0.08, 0.16)	.29* (0.20, 0.41)
SATAQ-General	−.18* (− 0.30, − 0,08)	.17* (0.04, 0.27)	.37* (0.26, 0.47)
Self-esteem	.78* (0.72, 0.83)	−.16* (− 0.28, − 0.05)	−.47*(− 0.57, − 0.39)
SWLS	.62* (0.53, 0.70)	−.19* (− 0.31, − 0.09)	−.35* (− 0.46, − 0.27)
PA h/w	.30* (0.20, 0.39)	.08 (− 0.03, 0.21)	−.01 (− 0.11, 0.09)
Exercise h/w	.29* (0.19, 0.38)	.07 (− 0.04, 0.18)	.06 (− 0.06, 0.18)
**Female students**			
BAS-2	1	−.17* (− 0.26, − 0.09)	−.56* (− 0.61, − 0.50)
Age	.01 (− 0.07, 0.08)	.04 (− 0.04, 0.15)	−.11* (− 0.19, − 0.03)
BMI	−.31* (− 0.40, − 0.23)	.08* (− 0.03, 0.16)	.17* (0.07, 0.25)
General experience of BAP	−.17* (− 0.26, − 0.09)	1	.42* (0.32, 0.46)
Personal experience of BAP	−.56* (− 0.61, − 0.50)	.42* (0.32, 0.46)	1
Comparison	−.66* (− 0.70, − 0.60)	.22* (0.13, 0.31)	.60* (0.53, 0.65)
SATAQ-Athletic	−.06 (− 0.14, 0.01)	.02(− 0.05, 0.11)	.16* (0.07, 0.25)
SATAQ-Thin	−.37* (− 0.45, − 0.32)	.21* (0.13, 0.29)	.44* (0.38, 0.51)
SATAQ-General	−.57* (− 0.64, − 0.52)	.13* (0.05, 0.22)	.47* (0.41, 0.53)
Self-esteem	.80* (0.77, 0.83)	−.16* (− 0.23, − 0.08)	−.46* (− 0.53, − 0.39)
SWLS	.57* (0.50, 0.64)	−.10* (− 0.17, − 0.01)	−.32* (− 0.40, − 0.24)
PA h/w	.11* (0.01, 0.22)	.00 (−0.09, − 0.08)	−.03 (− 0.13, − 0.05)
Exercise h/w	.15* (0.07, 0.23)	−.04 (− 0.11, 0.03)	−.01 (− 0.10, 0.07)

*Note*: University student sample analyzed as a whole, split on gender. Pearson Chi-square (r) results represent merged data from both exercise science and reference students, with corresponding 95% confidence interval (CI 95%) for correlations. BAS-2 = Body appreciation scale-2. BAP = body appearance pressure. Comparison = physical appearance comparison. SATAQ-4R = Social attitudes towards appearance questionnaire-4 revised with the male and female specific items. SWLS = Satisfaction with life scale. PA h/wk. = physical activity hours per week. Correlation is significant at the *0.05 (2-tailed)

We then performed correlational analyzes to evaluate association between BAP and other constructs. First, we found that in males and females, general experience of BAP was weakly correlated with some constructs. In both genders, we found that personal experience of BAP was moderately to strongly positively correlated with physical appearance comparison, general attractiveness internalization and internalization of the thin body ideal, in both genders, and the athletic body in males only. We also found that personal experience of BAP was moderately to strongly negatively correlated with body appreciation, self-esteem, and satisfaction with life, in males (Table 3, Fig. 1a) and females (Table 3, Fig. 1b).

Other constructs either showed weak or no significant correlation with body appreciation and/or personal experience of BAP for both genders (Table 3, Fig. 1).

## Discussion

This study aimed to explore body appreciation and the experiences of BAP in Norwegian university students of exercise science programs and to compare them with a reference group. We also aimed to explore the relationship between body appreciation, BAP, body image related mental health constructs, physical activity and exercise.

### Body appreciation

The mean scores on body appreciation found in both groups of males, and in female exercise science students, are comparable to what has been reported in male and female fitness instructors [10], but slightly higher than previously found in male and female dance students [27] and female undergraduate students [11]. These small differences between study samples might suggest that there could be cultural and context-related characteristics which may facilitate body appreciation more in exercise and fitness related settings, compared to the dance environment, which is known to be especially affected by focus on weight and shape [27]. It may also indicate that ordinary female students might not withhold the same well-known protective characteristics from physical activity engagement as exercise science and fitness instructors might do [7, 8].

The finding that female exercise science students reported a stronger body appreciation compared to reference students, supports similar differences found between adolescent students participating in leisure and competitive sports compared to non-participants [28]. Our results indicate that even though female exercise science students are exposed to a highly body-oriented environment and bodily capital, and report high levels of BAP, they also report characteristics that might protect their body image. In relation to previous studies focusing on negative body image related characteristics [16, 29, 30], our observation adds another dimension of exercise science students’ body image. Our finding could be explained by their higher levels of physical activity and exercise which could have had a positive influence on their body composition, leaving them more satisfied. This is in line with our finding that BMI influenced the variance in body appreciation explained by study program. The students’ high level of physical activity might additionally have facilitated an experience of body functionality, which alone is associated with body appreciation [9]. Also, the type of body focus throughout their studies, such as focusing on the body as a tool to engage in joyful activities, movement of the body to experience wellness and maintain health, may play a role in what value they put on different aspects of the body. Such focus might act as a positive enhancer of their body appreciation by enhancing the students neglection of body image threats, such as their experience of BAP [5].

### Experienced body appearance pressure

Despite the high levels of body appreciation reported by our sample, exercise science students experienced just as much BAP, and for males slightly more pressure, compared to other students. As such, BAP represents a challenge within the higher education system. Our study found that BAP was even more prevalent among Norwegian university students compared to the previously reported 70% in high school students [13], and more similar to ~ 90% found in Norwegian male and female fitness instructors [10]. Studying or working within an exercise environment with the suggested exposure to bodily capital may be seen in relationship to the high prevalence of BAP in these groups.

#### Exercise settings for body appearance pressure

In all student groups, fitness centers (commercial and at campus) were the most prevalent reported settings for exposure to BAP. This echoes findings by Mathisen et al. [10], and previous descriptions of fitness centers as a setting for reinforcement of body objectification and an appearance orientated environment [31]. Interestingly, more female reference students, compared to exercise science students, reported the fitness centers as the settings where they experienced the most BAP. Unfortunately, we did not measure body composition and fitness level, which could offer an opportunity to explore such differences between students. It could however, be reasonable to suggest that the reference students’ higher BMI and lower physical activity level, led to a stronger feeling of discrepancy between their body and the athletic body regularly displayed in these arenas, which might have affected BAP experience and associated mental health constructs [32].

### Associations between body appreciation, body appearance pressure, body image related mental health constructs, physical activity, and exercise

We found that body appreciation negatively corelated with physical comparison and internalization, and positively corelated with self-esteem and satisfaction with life. Our findings support previous observations among adolescents and young adults [6, 33, 34], and female college students [9]. Interestingly, female exercise science students reported a more favorable score on all these constructs compared to the reference students. This underlines the assumption that there is a positive relationship between physical activity engagement, body image related mental health variables, and body appreciation. This can further be understood through previous descriptions on how exercise engagement- and environments may be favorable to a range of mental health constructs and coping mechanisms [8, 35]. As such, the students’ physical activity level might not only be important for their body functionality experience, but to strengthen body appreciation and other mental health constructs that were found to associate with body appreciation.

The relationship between experienced BAP, engagement in physical appearance comparison, and internalization of body ideals, reflects, as suggested, the sociocultural model [32], and indicates that BAP is related to unfavorable cognitive processes of one’s body experience. This could relate to a preoccupation with the experience of how one’s body looks rather that the experience of its functionality, which could increase evaluation and comparison of one’s body appearance towards bodies that are idealized. Additionally, with the significant negative correlation to self-esteem and satisfaction with life, the personal experience of BAP is of great concern.

### Perspectives

Our study provides new information on how exercise sciences students differ in body image from other university students and adds a new dimension to our knowledge of exercise science students’ body image by measuring body appreciation. Also, the current study answered the questions related to prevalence of BAP and whether it did, as proposed, associate with health outcomes. Based on our findings, one might suggest that university students who are characterized with low levels of body appreciation, self-esteem, satisfaction with life, who often compare their physical appearance to others, and who strongly internalize appearance ideals, might be at higher risk of personally experiencing BAP. These students may benefit from being identified for further help. Since personal experienced BAP is prevalent among all students within our sample and is significantly related to an unfavorable pattern of body image related mental health constructs, actions need to be considered to reduce BAP among university students. Physical appearance comparison, internalization, self-esteem, and satisfaction with life all related to the experience of BAP and body appreciation, but in different directions. Also, BAP and body appreciation strongly correlated. Therefore, we may suggest a combined health promotion and risk reduction approach to make favorable changes in both constructs. Inclusion of body image related topics to prevent negative body image in university students within higher education programs has been recommended [36]. Media literacy could reduce students’ physical comparison level, their internalization, and possibly their personal experience of BAP [37]. Existing programs with such intervention components have been successful in improving self-esteem and body image in male and female trainee health education and physical education teachers [38], reducing thin-ideal internalization in female collegiate athletes [39], and body dissatisfaction and eating disorder risk factors in collegiate females [40]. Also, body functionality focused [41] and yoga interventions have been found effective in undergraduate females [42]. Based on current and previous findings related to fitness centers and BAP, actions are also needed within this environment to facilitate body appreciation through physical activity rather than BAP through body appearance focus. Importantly, longitudinal and experimental designs are needed to further explore the directional impact between body appreciation, BAP, and related mental health and behavioral constructs.

### Study strengths and limitations

The study provides novel information on body appreciation, BAP, and their associations, in a large sample of university students in exercise science programs compared with a reference group. Still, some limitations must be considered when interpreting our results. Our BAP instrument was not validated, as compared to the other measurements, and needs to be further evaluated. Also, self-report of weight, height, and physical activity level are important limitations, in addition to the anticipated low response rate. Finally, due to the cross-sectional study design, we are not able to discuss cause-effect.

## Conclusion

Body appreciation was found to be stronger among female exercise science students compared to reference students, while no group difference was observed in males. We identified a high level of BAP among all students, where fitness centers were reported as the most prevalent setting for BAP. When analyzing the sample as a whole, body appreciation was associated with favorable scores on body image related mental health constructs, while the opposite was found for BAP. Inclusion of body image topics early within education programs may play an important part in promoting body appreciation and preventing BAP among university students, and should be considered as essential for students’ health and well-being.

## Supplementary Information

**Additional file 1.**

## Data Availability

The datasets generated and analyzed during the current study are not publicly available due to further use of the datafile in upcoming studies but are available from the corresponding author on reasonable request.

## Abbreviations

BAP: Body appearance pressure
PA: Physical activity
BMI: Body mass index
BAS-2: Body Appreciation Scale, version 2
SATAQ-4R: Social attitudes towards appearance questionnaire, version 4 revised
PACS-R: Physical Appearance Comparison Scale-Revised
SWLS: Satisfaction with Life Scale
