# Supplementary material for: Body appreciation and body appearance pressure in Norwegian university students comparing exercise science students and other students
Source: BMC Public Health. 2021 Mar 19;21:532. doi: 10.1186/s12889-021-10550-0 (PMC7977603; doi:10.1186/s12889-021-10550-0)
Supplement: Supplementary file 1 — Additional file 1. [file 12889_2021_10550_MOESM1_ESM.docx]

| An English translation of the Body Appearance Pressure Scale  *We would like to ask you about your experience of, and opinions about, body appearance pressure. Read each question carefully and choose the response alternative that suits you best.*  Experience of body appearance pressure   1. *To what extent do you experience that the phenomenon body appearance pressure exists in general?* 2. Not at all 3. Some 4. To a high extent 5. To a very high extent 6. *To what extent do you personally experience body appearance pressure* 7. Not at all 8. Some 9. To a high extent 10. To a very high extent |
| --- |
| 1. *To what extent do you personally experience body appearance pressure in these different settings?* 2. *During lectures*    1. Not at all    2. Some    3. To a high extent    4. To a very high extent 3. *When you interact with fellow students*    1. Not at all    2. Some    3. To a high extent    4. To a very high extent 4. *When you interact with lectures*    1. Not at all    2. Some    3. To a high extent    4. To a very high extent 5. *At the fitness center connected to your campus/university*    1. Not at all    2. Some    3. To a high extent    4. To a very high extent    5. There is no fitness center connected to my campus 6. *At the fitness center not connected to the campus (commercial fitness centers)*    1. Not at all    2. Some    3. To a high extent    4. To a very high extent    5. I am not an active member at any fitness center 7. *Are there other settings than those mentioned, in which you experience body appearance pressure? Please specify:* __________________________________________________ |
| Body appearance pressure and bodily capital   1. *To what extent do you believe that having a specific body type results in benefits for the following examples?* 2. *General social recognition*    1. Not at all    2. Some    3. To a high extent    4. To a very high extent 3. *More followers on social media*    1. Not at all    2. Some    3. To a high extent    4. To a very high extent 4. *It plays an important role when recruiting clients for those who are employed as a personal trainers and/or physical activity and lifestyle coach*    1. Not at all    2. Some    3. To a high extent    4. To a very high extent 5. *Getting good grades in your study program*    1. Not at all    2. Some    3. To a high extent    4. To a very high extent 6. *Increases the chance of getting a job after graduation*    1. Not at all    2. Some    3. To a high extent    4. To a very high extent   Experienced consequences of body appearance pressure |
| 1. *To what extent does body appearance pressure affect you in different ways?* 2. *It affects me academically (e.g. the ability to study, concentrate, learn)*    1. Not at all    2. Some    3. To a high extent    4. To a very high extent 3. *It negatively affects my mood*    1. Not at all    2. Some    3. To a high extent    4. To a very high extent 4. *It affects me socially* (e.g. being out among people, with friends, in the way I function in the role as a friend, in a romantic relationship/as a family member)    1. Not at all    2. Some    3. To a high extent    4. To a very high extent 5. *It affects my self-esteem in a negative way (the way you experience yourself, who you are, what you believe in, the value you give yourself as you see yourself)*    1. Not at all    2. Some    3. To a high extent    4. To a very high extent 6. *It makes me try harder to meet the health authority’s physical activity and nutrition recommendations*     1. Not at all    2. Some    3. To a high extent    4. To a very high extent 7. *The pressure makes me exercise in a way that might not benefit my health and well-being, but in a way which I believe will affect the way I look*    1. Not at all    2. Some    3. To a high extent    4. To a very high extent 8. *The pressure makes me eat in a way that might not benefit my health and well-being, but in a way which I believe will affect the way I look* 9. Not at all 10. Some 11. To a high extent 12. To a very high extent   Measures to deal with body appearance pressure |
| 1. *Have you sought professional help (e.g. student health service, school nurse, social worker, psychologist, psychiatrist) to deal with body appearance pressure and the consequences it can result in?* 2. Yes, I am getting help now 3. Yes, I was given help previously 4. No, I have not considered it 5. No, but I would like to have a service where I could get help 6. No, I do not experience body appearance pressure as a challenge for me |
| 1. *Has your campus/university initiated specific actions/measures to reduce body appearance pressure?* 2. Yes 3. No 4. I don’t know   *If yes, what measure did they initiate?*  Please specify: _____________________________   1. *We would like to make the student life as good as possible, and to prevent body appearance pressure to have a negative impact on your time as a student. Which small or major measures do you believe could reduce the risk of experiencing body appearance pressure among students at your campus/university?*   *Please specify:* _________________________________________________________ |
